# Supplementary material for: Likely accelerated weakening of Atlantic overturning circulation emerges in optimal salinity fingerprint
Source: Nat Commun. 2023 Mar 4;14:1245. doi: 10.1038/s41467-023-36288-4 (PMC9985640; doi:10.1038/s41467-023-36288-4)
Supplement: Supplementary file 1 — supplementary information [file 41467_2023_36288_MOESM1_ESM.pdf]

Supplementary information for

Likely Accelerated Weakening of Atlantic Overturning Circulation Emerges in Optimal Salinity Fingerprint

Chenyu Zhu[1,2*], Zhengyu Liu[3,4*], Shaoqing Zhang[1,2*], Lixin Wu[1,2]

[1]Frontier Science Center for Deep Ocean Multispheres and Earth System (FDOMES) and Physical Oceanography Laboratory, Ocean University of China, Qingdao, China;
[2]Laoshan Laboratory, Qingdao, China
[3]Atmospheric Science Program, Department of Geography, Ohio State University, Columbus, OH 43210, USA
[4]College of Geography Science, Nanjing Normal University, Nanjing, China

*: Corresponding author: zhuouc@163.com (C.Z.); liu.7022@osu.edu (Z.L.); szhang@ouc.edu.cn (S.Z.)

The Supplementary information includes:

Supplementary Text
Figs. S1 to S7
Tables S1 to S2
Supplementary references

Supplementary Text

Decoupling between $(E\text{-}P)_s$ and Ss under anthropogenic forcing

We show that the change of S_S is not caused by the E-P under anthropogenic forcing. It is worth noting that well-mixed GHGs and heterogeneous aerosols both produce a global E-P signal, characterized by the strengthening (Fig.S5b) and weakening (Fig.S5e) of hydrographic cycle, respectively[1]. STSA shows relatively wetter (drier) condition compared with STSIP in response to increasing anthropogenic GHGs (aerosols), contributing negatively to the relatively greater salinification (freshening) in STSA SSS field (Fig.S5a,d and Fig.S4). Scatter plot across model members shows less consistency in sign between the relative change of E-P in STSA $(E\text{-}P)_S$ and S_S (Fig.S5c,f), in contrast to the strong consistency between AMOC and S_S (Fig.3e,f). The basin-mean budget shows more clearly that while S_{SA} has some consistency in sign with the local E-P change $(E\text{-}P)_{SA}$ (Fig.S4c,d), the S_S change, which is very similar to that of S_S, is independent from the relative (E-P) change $(E\text{-}P)_S$, especially for forced experiments hist-GHG and hist-aer (Fig.S4a,b). This implies a dominant impact of AMOC change on both S_S and S_{SA} over the atmospheric hydrological impact, consistent with the investigation of RCP scenarios and the ocean sensitivity experiments in *ref.*[2]. The dominant role of ocean circulation change has also been investigated with salinity budget analysis in both coupled and ocean alone simulations[2].

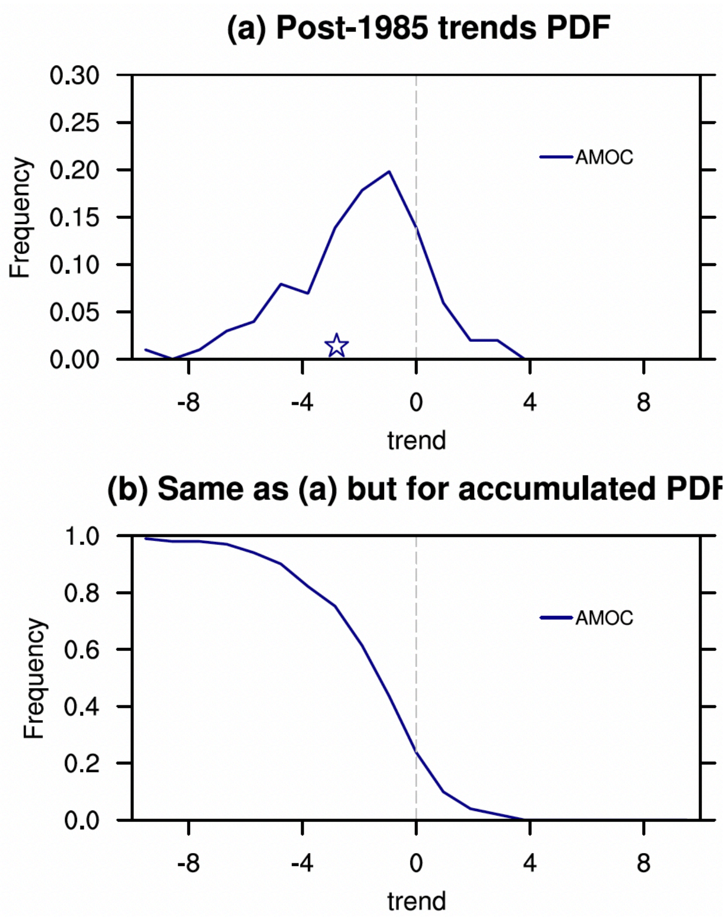

Fig. S1. Post-1980s trends of Atlantic meridional overturning circulation (AMOC) intensity. (a) AMOC trends (Sv/30yr) distribution shown as a probability density function (PDF) in the combined CMIP5 and CMIP6 model members (101 members). **(b)** Same as (a) but for accumulated PDF. Star is the trend of ECCO AMOC.

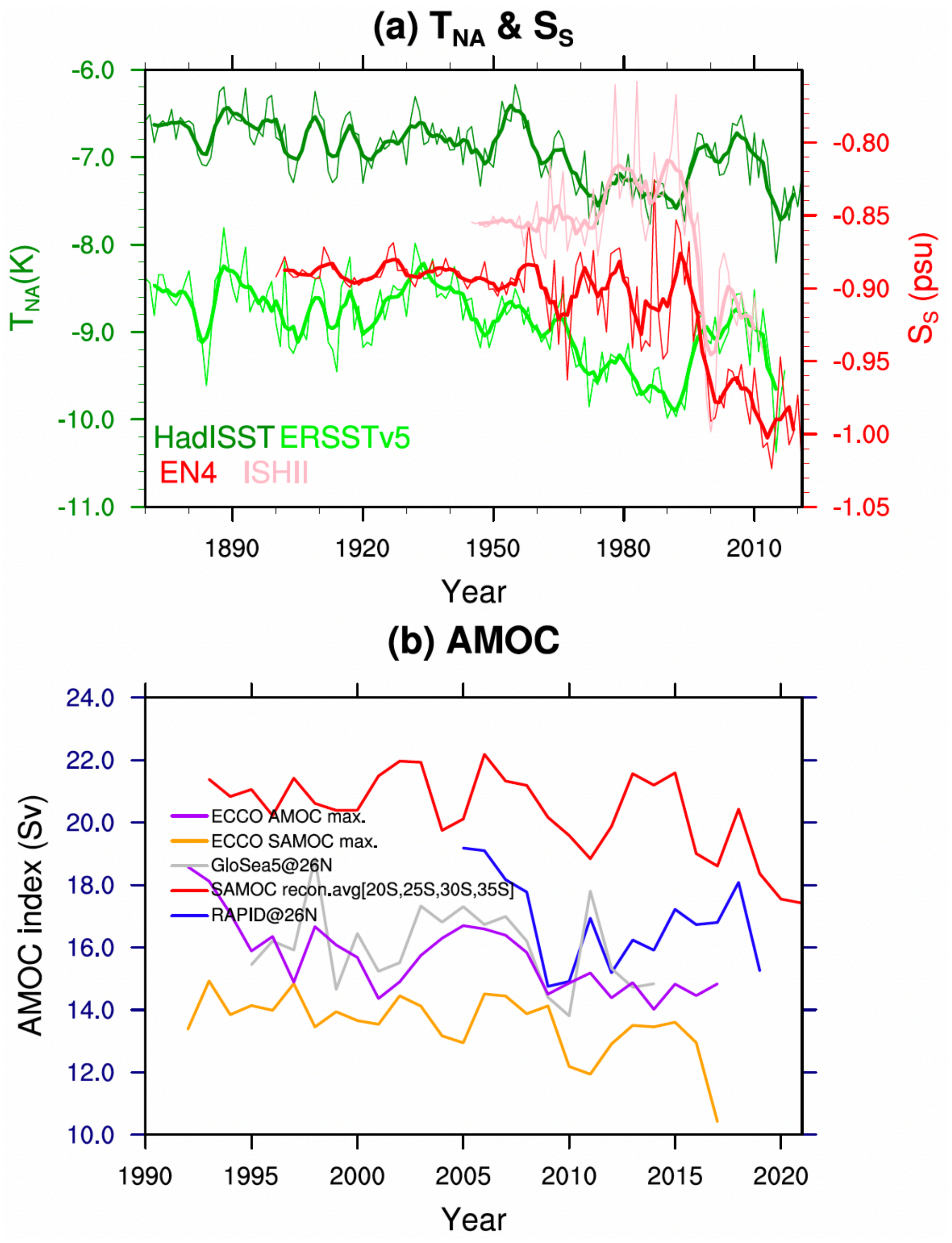

Fig. S2. Comparison of indices derived from different observational datasets. (**a**) salinity-based AMOC fingerprint S_S and warming hole fingerprint T_{NA}; (**b**) AMOC index. Thin and Thick lines in (**a**) are for annual and 5-yr running mean time series, respectively.

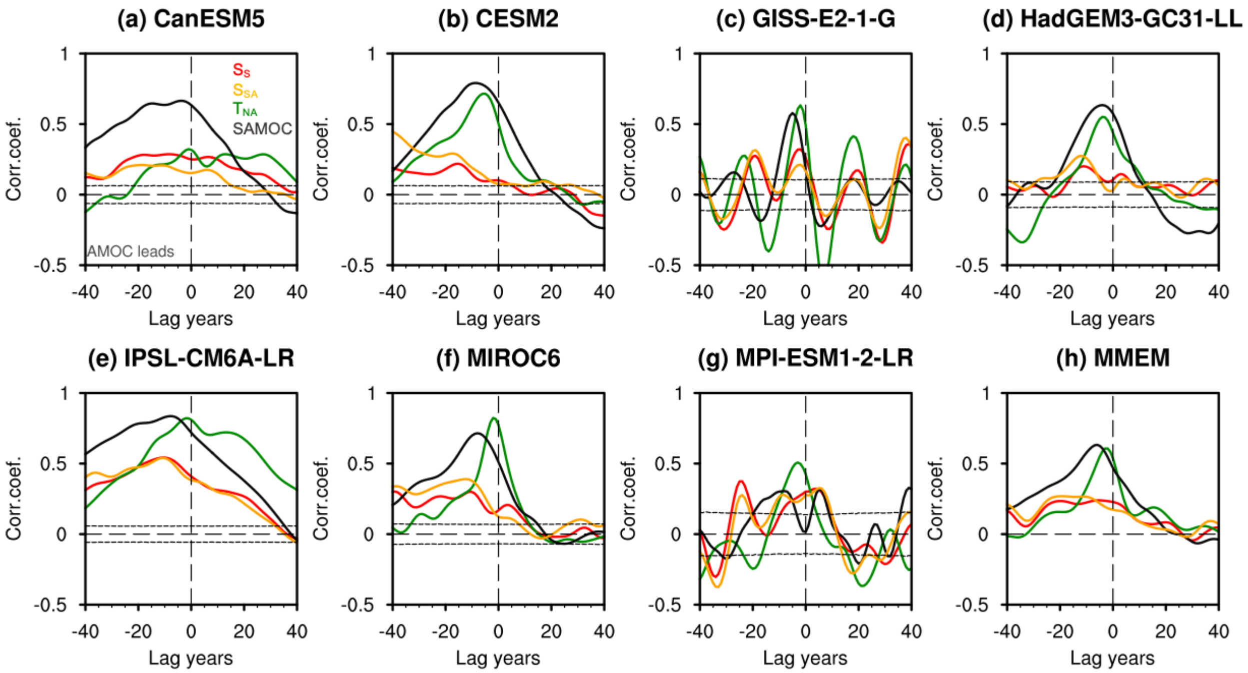

Fig. S3. Lead-lag correlation for piControl in each model. **(a)** CanESM5, **(b)** CESM2, **(c)** GISS-E2-1-G, **(d)** HadGEM3-GC31-LL, **(e)** IPSL-CM6A-LR, **(f)** MIROC6, **(g)** MPI-ESM1-2-LR and **(h)** Multi-model ensemble mean (MMEM), the same as Fig.3(**a**). Short dashed lines indicate the 95% significance level determined using Monte Carlo method.

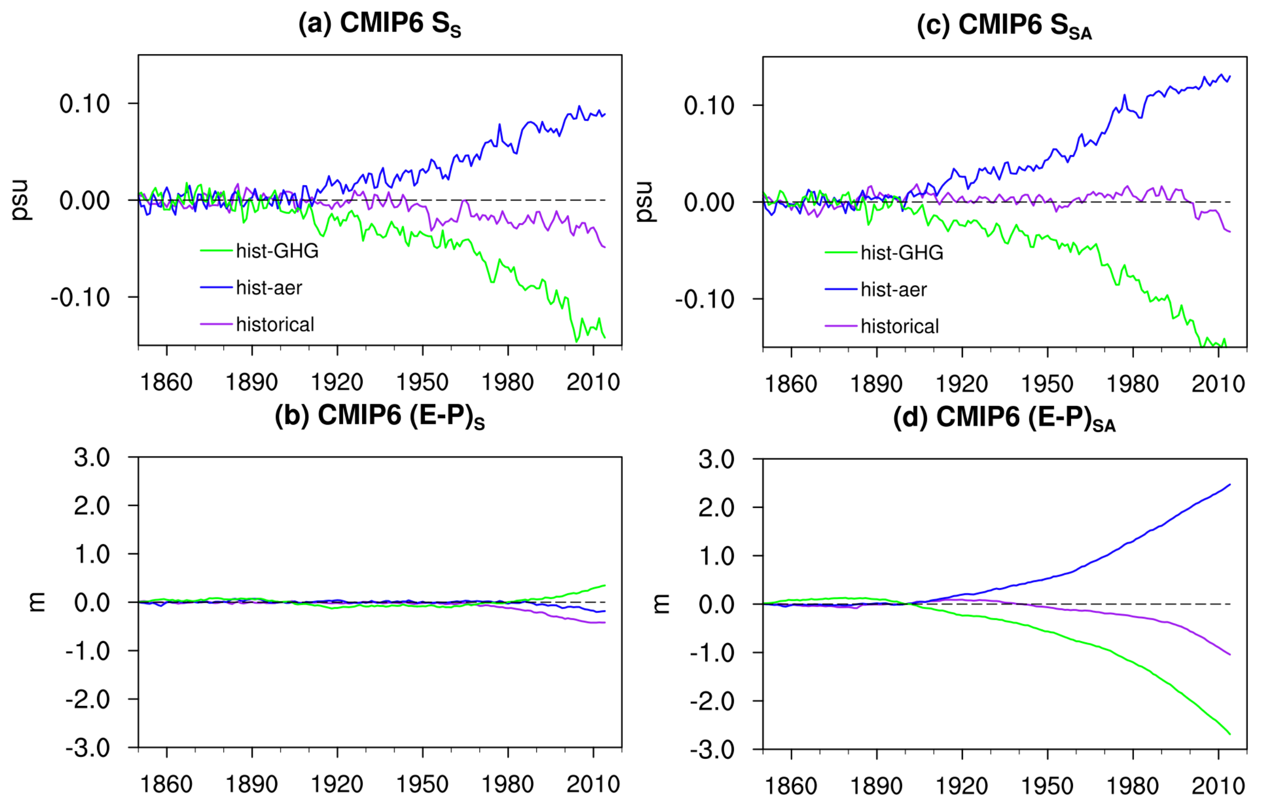

Fig. S4. Response of salinity and (E-P) in historical, Hist-GHG and Hist-aer simulations. (a) S_S, **(b)** $(E\text{-}P)_S$, **(c)** S_{SA} and **(d)** $(E\text{-}P)_{SA}$. Anomalies are relative to the means of 1850-1900. Note the neglectable role of $(E\text{-}P)_S$ on S_S especially under GHGs and aerosol forcing. Here $(E\text{-}P)_s$ is the (E-P) change of STSA relative to STSIP, defined similar to S_S.

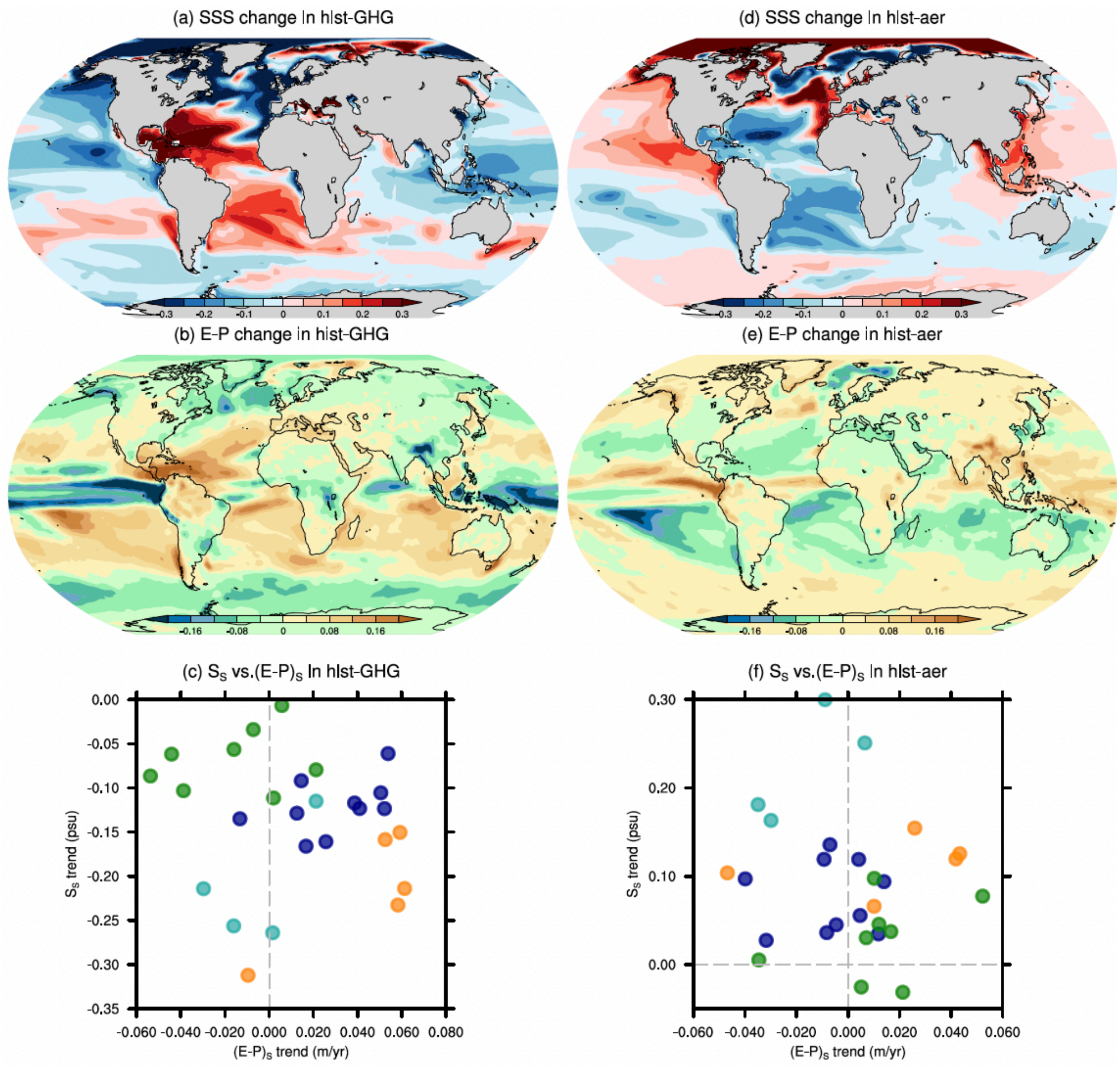

Fig. S5. Response of E-P and SSS in Hist-GHG and Hist-aer. (**a**) linear changes of SSS (psu) between 1900 and 2014 in Hist-GHG;(**b**) same as (**a**) but for (E-P)(m/yr); (**c**) scatter plots of S_S trends and (E-P)$_S$ trends across model members in Hist-GHGs . (**d,e,f**) same as (**a,b,c**) but for hist-aer simulation. Note the opposing phase between relative SSS and (E-P) change over STSA compared with STSIP (**a** vs. **b**, **d** vs. **e**). See Fig.2 for color convention of CMIP6-DAMIP model (Table S2).

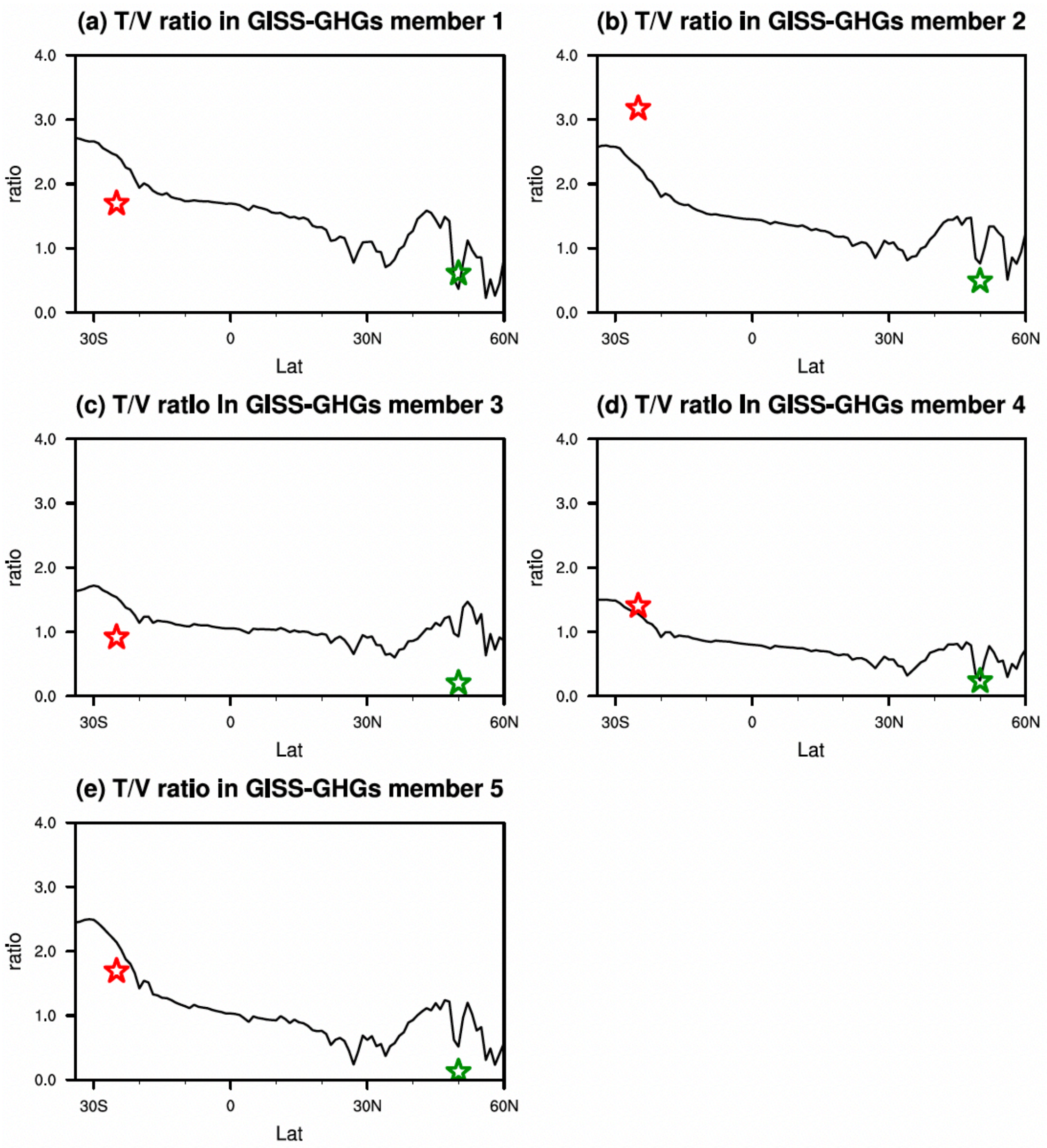

Fig. S6. (a, b, c, d, e) show trend/variability ratio of AMOC (black line), salinity pileup index (red star) and warming hole index (green star) for each GISS-GHGs ensemble member.

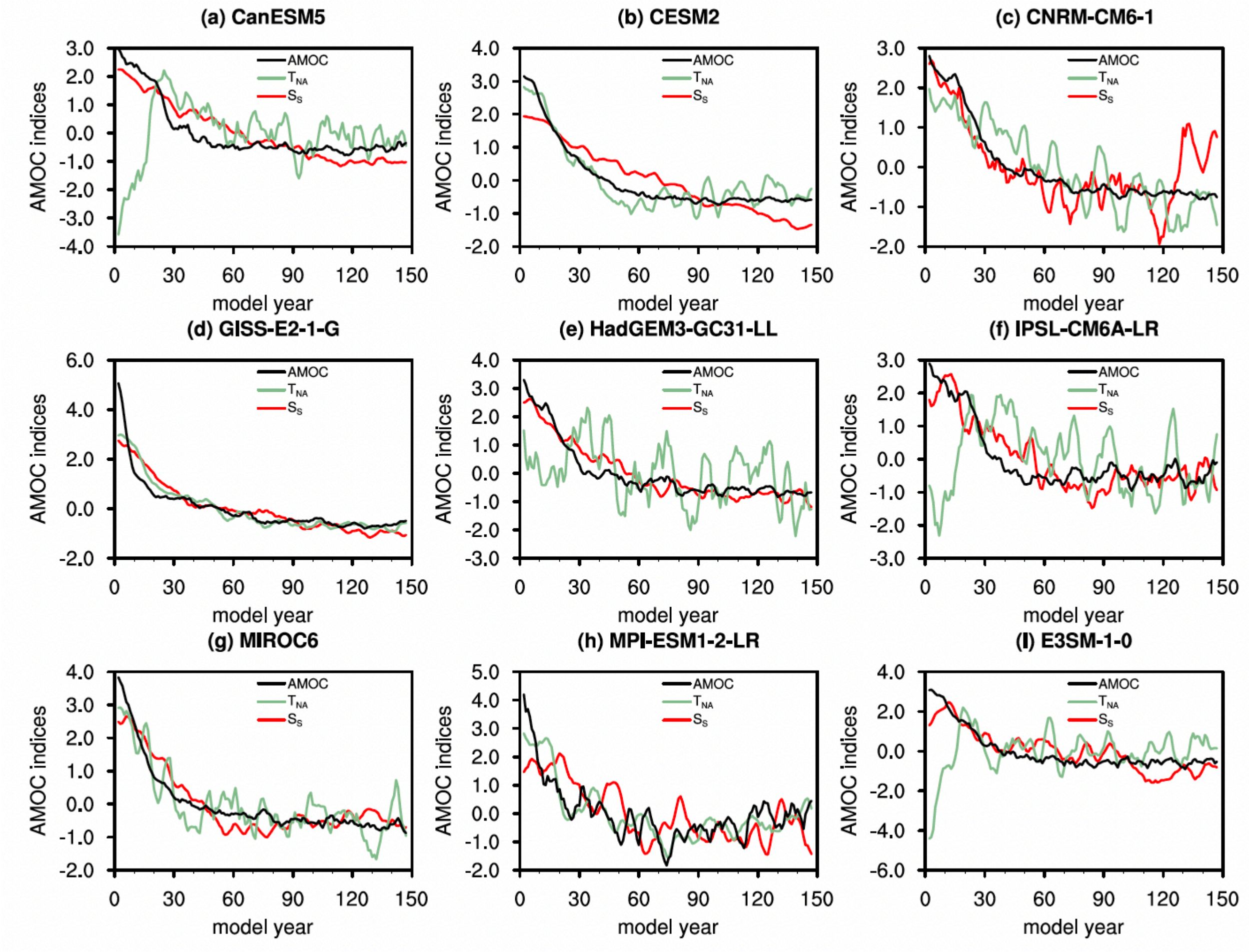

Fig. S7. **Response of Atlantic meridional overturning circulation (AMOC) indices to abrupt forcing in different models**. **(a)** CanESM5, **(b)** CESM2, **(c)** CNRM-CM6-1, **(d)** GISS-E2-1-G, **(e)** HadGEM3-GC31-LL, **(f)** IPSL-CM6A-LR, **(g)** MIROC6, **(h)** MPI-ESM1-2-LR, **(I)** E3SM-1-0. Change of AMOC (black), warming hole fingerprint T_{NA} (green) and salinity pileup fingerprint S_S (red) to the abrupt CO_2 quadrupling in 8 CMIP6 models and another CGCM of E3SM1.0. All indices are after 5-year running mean and normalized by their standard deviation with their mean as zero.

Table S1. CMIP models. CMIP5 and CMIP6 models used in the investigation of all-forcing historical simulation, PI control simulation and abrupt CO_2 quadrupling simulation.

Model		Historical ensemble	PI ensemble (length)	Abrupt-4xCO_2 (length)
CMIP5	CanESM2	5		
	CCSM4	4		
	CESM1-CAM5	3		
	CNRM-CM5	5		
	GISS-E2-R	5		
	MPI-ESM-LR	3		
	MPI-ESM-MR	3		
	NorESM1-M	3		
	Total models (ensemble members)	8 (31)		
CMIP6	CanESM5	10	1(1000 years)	1(150 years)
	CESM2	10	1(999 years)	1(150 years)
	CNRM-CM6-1	6		1(150 years)
	GISS-E2-1-G	10	1(345 years)	1(150 years)
	HadGEM3-GC31-LL	4	1(500 years)	1(150 years)
	IPSL-CM6A-LR	10	1 (1200 years)	1(150 years)
	MIROC6	10	1 (800 years)	1(150 years)
	MPI-ESM1-2-LR	10	1 (200 years)	1(150 years)
	Total models (ensemble members)	8 (70)	7 (7)	7(7)

Table S2. DAMIP experiments. CMIP6-DAMIP experiments used in the investigation of single-forcing simulation.

DAMIP Model	Hist-aer	Hist-nat	Hist-GHG	Hist-stratO3
CanESM5	10	10	10	10
CNRM-CM6-1	9	9	9	
GISS-E2-1-G	5	5	5	5
HadGEM3-GC31-LL	4	4	4	
IPSL-CM6A-LR	8	8	8	8
Total models (ensemble members)	5(36)	5(36)	5(36)	3(23)

Supplementary references

1. Xie, S. P., Lu, B., Xiang, B. Similar spatial patterns of climate responses to aerosol and greenhouse gas changes. *Nature Geosci.* **6**, 828-832 (2013).
2. Zhu, C., Liu, Z. Weakening Atlantic overturning circulation causes South Atlantic salinity pile-up. *Nature Climate Change* **10**, 998-1003 (2020).
